# Underlying Disease and Clinical Phenotypes of Disseminated Intravascular Coagulation

**DOI:** 10.31662/jmaj.2020-0072

**Published:** 2020-10-02

**Authors:** Kensuke Matsuda, Mineo Kurokawa

**Affiliations:** 1 Department of Hematology and Oncology, Graduate School of Medicine, University of Tokyo, Tokyo, Japan

**Keywords:** disseminated intravascular coagulation, clinical phenotypes, recombinant thrombomodulin

Disseminated intravascular coagulation (DIC) is a life-threatening condition of sustained, widespread coagulation activation in the presence of severe underlying disease ^(1)^. Based on its characteristics, DIC can be classified into three disease types. The first is DIC with suppressed fibrinolysis, which is frequently observed in sepsis and associated with organ failure. It is characterized by an abnormal increase in coagulation activation. The second is DIC with increased fibrinolysis, which is usually seen in aortic disease and acute promyelocytic leukemia (APL). Fibrinolysis is abnormally activated, and fatal bleeding are occasionally observed. Third, DIC with balanced fibrinolysis, which is a type of DIC observed in solid tumors and is intermediate between the two aforementioned types. To manage the clinical symptoms of DIC, appropriate interventions should be performed according to these three types of DIC.

Ohbe H, et al. performed a nationwide analysis on the relationship between underlying disease and clinical DIC phenotype ^(2)^. The current study analyzed >300,000 patients with DIC and various underlying diseases including sepsis, aortic disease, leukemia, and solid tumor. The results showed that the clinical presentations of bleeding and organ failure are not associated with the three existing clinical phenotypes of DIC. Although they did not evaluate the laboratory data including plasma fibrinogen levels in the current study, the results showed that a novel indicator may be necessary to predict clinical manifestations of DIC.

The management of DIC generally includes three approaches ^(3)^. First, treatment of the underlying disease is the most important approach. Because the underlying diseases can trigger DIC, DIC cannot be treated without controlling the underlying condition. Second, the transfusion of platelets or fresh frozen plasma may be required depending on laboratory findings and physical conditions. Finally, although the effectiveness continues to be controversial, anticoagulant therapy including heparin and thrombomodulin is also available.

Recombinant thrombomodulin is widely used in Japan. Certain reports showed the effectiveness of recombinant thrombomodulin in patients with sepsis and leukemia, but comprehensive analysis of various diseases did not confirm the effectiveness. Considering the results of the current study, the use of anticoagulants should not be decided based on the underlying disease but on the actual DIC phenotypes.

The management of DIC has improved with the results of many studies. However, a considerable amount of information remains unclear regarding the pathogenesis and management of DIC, and further research is warranted. In addition, more efforts should be taken to improve the awareness of DIC among healthcare providers because early intervention may improve the outcomes.

## Article Information

### Conflicts of Interest

Mineo Kurokawa received lecture fees from Asahi Kasei Corporation.

### Disclaimer

Mineo Kurokawa is one of the Editors of JMA Journal and on the journal's Editorial Staff. He was not involved in the editorial evaluation or decision to accept this article for publication at all.
